# Minimal Intensity Physical Activity (Standing and Walking) of Longer Duration Improves Insulin Action and Plasma Lipids More than Shorter Periods of Moderate to Vigorous Exercise (Cycling) in Sedentary Subjects When Energy Expenditure Is Comparable

**DOI:** 10.1371/journal.pone.0055542

**Published:** 2013-02-13

**Authors:** Bernard M. F. M. Duvivier, Nicolaas C. Schaper, Michelle A. Bremers, Glenn van Crombrugge, Paul P. C. A. Menheere, Marleen Kars, Hans H. C. M. Savelberg

**Affiliations:** 1 Department of Internal Medicine, Maastricht University Medical Centre, Maastricht, The Netherlands; 2 Department of Human Movement Sciences, Maastricht University Medical Centre, Maastricht, The Netherlands; 3 Central Diagnostic Laboratory, Maastricht University Medical Centre, Maastricht, The Netherlands; Institut Pluridisciplinaire Hubert Curien, France

## Abstract

**Background:**

Epidemiological studies suggest that excessive sitting time is associated with increased health risk, independent of the performance of exercise. We hypothesized that a daily bout of exercise cannot compensate the negative effects of inactivity during the rest of the day on insulin sensitivity and plasma lipids.

**Methodology/Principal Findings:**

Eighteen healthy subjects, age 21±2 year, BMI 22.6±2.6 kgm^−2^ followed randomly three physical activity regimes for four days. Participants were instructed to sit 14 hr/day (sitting regime); to sit 13 hr/day and to substitute 1 hr of sitting with vigorous exercise 1 hr (exercise regime); to substitute 6 hrs sitting with 4 hr walking and 2 hr standing (minimal intensity physical activity (PA) regime). The sitting and exercise regime had comparable numbers of sitting hours; the exercise and minimal intensity PA regime had the same daily energy expenditure. PA was assessed continuously by an activity monitor (ActivPAL) and a diary. Measurements of insulin sensitivity (oral glucose tolerance test, OGTT) and plasma lipids were performed in the fasting state, the morning after the 4 days of each regime. In the sitting regime, daily energy expenditure was about 500 kcal lower than in both other regimes. Area under the curve for insulin during OGTT was significantly lower after the minimal intensity PA regime compared to both sitting and exercise regimes 6727.3±4329.4 vs 7752.0±3014.4 and 8320.4±5383.7 mU•min/ml, respectively. Triglycerides, non-HDL cholesterol and apolipoprotein B plasma levels improved significantly in the minimal intensity PA regime compared to sitting and showed non-significant trends for improvement compared to exercise.

**Conclusions:**

One hour of daily physical exercise cannot compensate the negative effects of inactivity on insulin level and plasma lipids if the rest of the day is spent sitting. Reducing inactivity by increasing the time spent walking/standing is more effective than one hour of physical exercise, when energy expenditure is kept constant.

## Introduction

Balancing energy intake and expenditure is the current paradigm in promoting lifestyle related health behaviour and is the basis for many physical activity (PA) guidelines [1]. From the point of thermodynamics this focus is understandable and it is usually assumed that the beneficial effects of PA increase in parallel to its intensity, ‘the more the better’. However, evidence is growing that sedentary time is a health risk factor on its own, independent of the practice of exercise. Television viewing time or sitting time in general is associated with increased mortality in epidemiological studies [2]. As reviewed elsewhere there is some evidence for a positive relationship between sitting time and the risk of type 2 diabetes [3]. Experimental data from studies in rodents [4], as well as data from cross-sectional studies in humans [5], [6], [7], suggest that excessive sitting time is associated with adverse changes in circulating lipids and insulin sensitivity. Recent intervention studies also showed that short-term reduction of daily PA negatively affects insulin sensitivity. Reducing habitual physical activity during 2 weeks to approximately 15% resulted in a 17% decline of glucose infusion rate in a hyperinsulinemic-euglycemic clamp procedure [8]. In another more acute experiment, energy expenditure was reduced to ∼75% of the normal level during 24 hr, with and without a compensatory decrease in energy intake [9]. In the condition where energy intake was not decreased, insulin sensitivity was 39% lower; when the reduction in energy expenditure was compensated with a decrease in energy intake, insulin sensitivity was reduced by 18%. These data suggest that inactivity may have negative effects on insulin sensitivity independent of energy balance.

Insulin resistance is thought to play a central role in the development of type 2 diabetes. Several lines of evidence indicate that physical inactivity can lead to skeletal muscle insulin resistance and possibly to lipid abnormalities [4], [5], [6], [7]. Moderate to vigorous PA can markedly improve the metabolic consequences of a sedentary lifestyle, by increasing daily energy expenditure (DEE) and augmenting muscle insulin signaling [10]. Several research groups have shown that regular exercise can prevent type 2 diabetes and current guidelines recommend at least 150 minutes/week of moderate to vigorous PA [11]. Unfortunately, in our society many adults do not reach this activity goal [12]. Moreover, current guidelines provide no guidance how, besides the 150 minutes of moderate to vigorous PA/week, the other 9930 minutes of the week should be spent. In the present study we tested the hypothesis that the negative metabolic effects of excessive sitting cannot be compensated by 1 hour of daily physical exercise. We used activity monitors that measure 24/7 energy expenditure and posture allocation, enabling us to distinguish the effects of sedentarism from minimal daily physical activities. Under free living conditions, sitting time, physical exercise and daily energy expenditure were manipulated in healthy volunteers in three well controlled experimental conditions in order to determine the independent effects of excessive sitting on insulin sensitivity and circulating lipids.

## Materials and Methods

### Subjects

Twenty healthy volunteers (students of the Maastricht University, 17 females and 3 males) were recruited via advertisement. To be included in the study, participants had to perform physical exercise less than 1 hr/week, their BMI should be between 20–30 kg/m^2^ and their age between 18 to 30 years. Exclusion criteria were any drug use (except oral contraceptives); diseases which interfered with physical activities; frequent alcohol use (more than two units/day); fasting triglycerides >3.0 mmol/l and a fasting plasma glucose >6.0 mmol/l. The study complied with the Declaration of Helsinki and was approved by the Local Ethics Committee of Maastricht University Medical Centre; all participants gave written informed consent. The study was registered as NCT01299311 at ClinicalTrials.gov.

### Study Design

**Figure 1:**
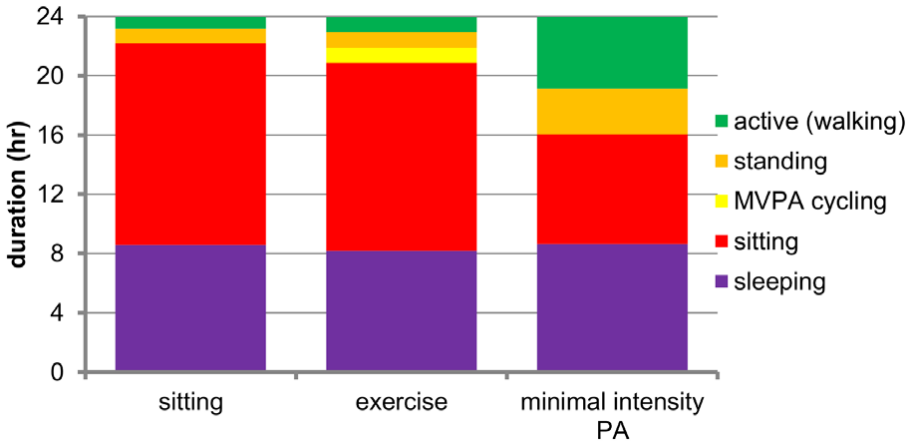
Time spent on different activities per regime. Graphical overview of time spent in different activity categories (sleeping, sitting, standing, MVPA cycling and activity (walking)) in the three regimes followed by the participants.

The study was performed under free living conditions and all participants were instructed to follow three activity regimes of four days each. A counterbalanced, randomised crossover design was used, in which participants served as their own controls (Figure 1). In the sitting regime subjects were instructed to sit 14 hr/day, to walk 1 hr/day, to stand 1 hr/day and to spend 8 hr/day sleeping or supine. In the exercise regime 1 hr of sitting was replaced by 1 hr vigorous supervised bicycling per day, the rest of the day was spent similarly as during the sitting regime. In the minimal intensity PA regime subjects were instructed to replace 6 hr of sitting with 4 hr of walking at a leisure pace and with 2 hr of standing. The sitting and exercise regime had only 1 hr difference in sedentary behaviour, but had considerably different energy expenditure. The exercise and minimal intensity PA regimes differed largely in time spent sitting or lying but were designed to have comparable energy expenditure. The intensity/duration of the physical exercise and duration of extra standing/walking during the exercise and minimal intensity PA regimes were chosen to result in the same increase in DEE (450 kcal) compared to the sitting regime. The order of regimes was randomised. Besides vigorous cycling for 1 hr/day during the exercise regime, any other kind of exercise was not allowed. Between every activity regime a washout period of at least 10 days was scheduled. Subjects were asked to maintain their usual pattern of daily activities during these washout periods.

Subjects were instructed to consume the same caloric intake during each regime and to maintain their usual dietary habits during the three activity regimes but dietary intake was not controlled, e.g. by providing meals or food ingredients. Subjects were not restricted in foods consumed except that they were asked to refrain from alcohol. During each activity regime participants kept a food diary in which daily intake was entered and after each activity regime they filled out a questionnaire on changes in health, medication and impact of the study on daily activities.

### Assessment of Physical Activity, Postural Allocation and Energy Expenditure

During the four days of a regime participants wore continuously (24 hours a day) an ActivPAL™ activity monitor (PAL Technologies, Glasgow, Scotland) to quantify daily PA and postural allocation. The monitor was attached to the skin on the anterior aspect of the thigh using Tegaderm (3M™); non-wear was therefore not an issue. Waterproof wrapping of the monitors allowed wearing during water activities such as bathing. This accelerometer-based activity monitor discriminates time spent sitting or lying, standing and active. In addition stepping bouts and activity intensity were determined and energy expenditure was estimated. Validity and reliability of the ActivPAL in assessing activity pattern of free living healthy subjects has been shown previously [13]. In addition, participants reported in a diary every 15 minutes the time spent walking, standing and/or sitting during waking hours using a stopwatch. To ensure that the daily activities were according to the study protocol, all subjects performed a run-in day before the start of the activity periods; the activity pattern recorded on that day was used to formulate tailor-made instructions on how to change daily activities during the different regimes. Moreover, the ActivPAL data were evaluated and discussed after the first day of each regime; subsequently subjects mailed their diary data every day and received electronic advice on their daily activities and posture allocation.

In the exercise regime, participants cycled for 1 hr at Maastricht University Medical Centre^+^ on a bicycle ergometer (Bodyguard cardiocycle 975). To control intensity and energy expenditure of cycling the participants’ heart rate was monitored continuously (Polar, Kempele, Finland). The model by Hiiloskorpi et al. [14] was used to calculate for each individual the heart rate that corresponded with 450 kcal energy expenditure.

Based on the diaries the sleeping time was determined. To calculate sitting time the sleeping time was subtracted from the ActivPAL class ‘sitting/lying’. In addition to the posture allocation, the ActivPAL assessed energy expenditure as metabolic equivalents (MET). By multiplying MET-values by estimated basal metabolic rate (BMR, Harris-Benedict equation), estimated energy expenditure as kcal was obtained. For the exercise condition 450 kcal spent cycling was added. Data of posture allocation and energy expenditure were averaged over four days for each regime.

### Insulin Sensitivity and Lipid Metabolism Assessment

Measurements of insulin sensitivity (oral glucose tolerance test, OGTT) and plasma lipids were performed in the fasting state, the morning after the 4 days of each activity regime at the Clinical and Translational Research Centre facility. The OGTT was chosen as a measure for insulin sensitivity because of its relative simplicity enabling a large number of measurements and its acceptable correlation with the gold standard (i.e. hyperinsulinemic euglycemic clamp). The minimum time between the last bicycle exercise bout during the exercise regime and the OGTT was 16 hours (mean interval 20±2.6hours). An i.v. catheter was placed in an antecubital vein for blood sampling. At baseline blood was sampled for analysis of glucose, insulin, C-peptide, triglycerides, total cholesterol, high- (HDL-C) and low-density-lipoprotein cholesterol (LDL-C), non-HDL cholesterol, apolipoprotein A-I and B (apo A-I and apo B). After ingestion of 75 g of glucose in 250 ml of water, blood samples were drawn for glucose, insulin and C-peptide levels at 15, 30, 45, 60, 90, and 120 minutes.

Blood samples for glucose, total cholesterol, HDL-C, LDL-C, non-HDL cholesterol and triglycerides were determined the same day. Samples for insulin, C-peptide, apo A-I and apo B were stored at −20°C until analysis after the end of the study. Plasma glucose, total cholesterol, HDL-C, triglycerides were colometric analysed on a Synchron LX20 Pro (Beckman Coulter). Insulin was measured with a double antibody radioimmunoassay Auto-Delfia (Perkin Elmer) and C-peptide with a double chemiluminiscent immunometric Immulite 2000 (Siemens). Apo A-I and apo B were nefelometric determined with a BN ProSpec (Siemens). LDL-C was calculated using the Friedewald formula [15].

### Statistical Analysis and Calculations

If in the series of seven OGTT sample points one or two values missed, polynomial regression was used to assess the best fitting second or third degree polynomial through the available sample points. The best fitting polynomial was used to determine the missing sample points. For each of the OGTT measurement intervals, the product of the duration of the interval and the average insulin, glucose and C-peptide level respectively was calculated. The area under the curve for insulin, glucose and C-peptide curves for the 2 hour period of the OGTT was calculated as the sum of these intervals. As a measure of insulin sensitivity, the insulin sensitivity index (ISI) was assessed [16].

All statistical analyses were executed with SPSS (SPSS 18, Chicago, IL, USA). Values are reported as mean±standard deviations. Variables were tested for normality and homogeneity. Repeated measures ANOVA was applied to evaluate the influence of the different regimes on plasma lipids, on areas under the curve (AUC) of insulin, glucose and C-peptide and on ISI. P-values of ≤0.05 were considered statistically significant. If the repeated measures ANOVA revealed a statistically significant effect of the intervention, conditions were pairwise compared using a Least Significant Difference (LSD) test. Since the LSD test does not correct for multiple testing, only p-values less than 0.017 (0.05/3) were considered significant in the pairwise comparison. To test whether changes in insulin sensitivity were associated with adaptations in plasma lipids, Pearson’s correlation coefficients were calculated between changes in triglyceride concentration over the regimes and changes in ISI.

## Results

Two subjects (one male, one female) withdrew before completing the protocol. The participants were on average 21 years of age, had a normal BMI with normal plasma lipid and glucose values (Table 1).

**Table 1:** Subject characteristics.

Variables	Means ± SD
**N**	18
**Age (years)**	21±2
**Height (m)**	1.68±0.07
**Weight (kg)**	63.9±7.8
**BMI (kg/m^2^)**	22.6±2.6
**Fasting glucose** a **(mmol/l)**	4.61±0.31
**Total cholesterol** a **(mmol/l)**	4.64±0.70
**Triglycerides** a **(mmol/l)**	0.89±0.25
**HDL-cholesterol** a **(mmol/l)**	1.45±0.34
**LDL-cholesterol** a **(mmol/l)**	2.77±0.56

a n = 17.

### Physical Activity, Postural Allocation and Energy Intake and Expenditure

The number of hours slept did not differ between the regimes and the study succeeded in manipulating independently inactivity time, walking/standing time and physical exercise (Table 2). During the exercise regime all participants had a daily, 1 hour bicycle exercise with a mean increase of heart rate of 52±3 beats/min, resulting in an estimated energy expenditure of 453±10 kcal. Compared to the sitting regime the time spent active (i.e. not sitting) but not exercising was somewhat higher during the exercise regime, with approximately an extra of 1700 steps/day (table 2). Standing (∼2 hours) and walking time (almost 4 hours) were markedly increased during the minimal intensity PA regime compared to both other regimes; consequently the number of steps was 5 to 6 times higher during this regime (Table 2). Based on the 24 hr ActivPAL data, energy expenditure during walking in the minimal intensity PA regime was estimated to equal an average of ∼3 METs; this is classified as light intense physical activity [17]. Compared to the sitting regime, estimated DEE was about 500 kcal higher during both other regimes; estimated DEE was 73 kcal/day higher during the minimal intensity PA in comparison to the exercise regime: 2407 vs 2486 kcal/day (p = 0.022). The self-reported caloric intake and the macronutrient composition did not differ between the regimes.

**Table 2:** Daily energy intake and expenditure, time spent in activity categories and glucose metabolism and plasma lipids.

	Sitting regime	Exercise regime	Minimal intensityPA regime	p-value	p sit vs exerc.	p sit vsMIPA	p exerc vs MIPA
**Daily Energy intake (kcal, n = 18)**	1539(427)	1477(352)	1394(292)	0.136			
**Proteins (g, n = 18)**	61.1(14.8)	59.7(13.5)	55.6(13.4)	0.165			
**Fat (g, n = 18)**	54.5(14.7)	50.2(19.6)	50.1(12.2)	0.248			
**Carbohydrates (g, n = 18)**	199.0(68.9)	196.7(48.9)	180.0(51.2)	0.227			
**Daily Energy Expenditure (kcal, n = 16)**	1934(88)	2407(100)	2486(121)	<0.001	<0.001	<0.001	0.022
**Sitting time (hr, n = 17)**	13.6(1.2)	12.7(1.7)	7.4(1.3)	<0.001	0.002	<0.001	<0.001
**Standing time (hr, n = 17)**	0.99(0.50)	1.08(0.48)	3.08(0.88)	<0.001	0.166	<0.001	<0.001
**Active-not exercise time (hr, n = 17)**	0.81(0.29)	1.01(0.26)	4.85(0.63)	<0.001	0.001	<0.001	<0.001
**Sleeping time (hr, n = 17)**	8.58(0.74)	8.17(1.37)	8.65(0.93)	0.200			
**Steps/day (n = 16)**	4324(1485)	6049(1402)	27590(3724)	<0.001	<0.001	<0.001	<0.001
**Triglycerides (mmol/l; n = 18)**	0.90(0.26)	0.85(0.35)	0.70(0.23)	0.007	0.326	0.002	0.029
**Total Cholesterol (mmol/l; n = 18)**	4.20(0.67)	4.11(0.60)	3.96(0.50)	0.171			
**HDL-Cholesterol (mmol/l; n = 18)**	1.26(0.34)	1.27(0.28)	1.30(0.30)	0.686			
**Non-HDL-Cholesterol (mmol/l; n = 18)**	2.94(0.47)	2.84(0.57)	2.65(0.48)	0.011	0.275	0.007	0.048
**LDL-Cholesterol (mmol/l; n = 18)**	2.53(0.51)	2.45(0.57)	2.34(0.49)	0.094			
**Apo A-I (g/l; n = 18)**	1.57(0.24)	1.57(0.21)	1.55(0.21)	0.905			
**Apo B (g/l; n = 18)**	0.75(0.12)	0.70(0.16)	0.69(0.14)	0.022	0.052	0.005	0.627
**Insulin Sensitivity Index (n = 17)**	20.4(8.2)	22.8(9.9)	26.3(11.7)	0.052	0.246	0.051	0.036
**Fasting Glucose** **(mmol/ml; n = 17)**	4.6(0.4)	4.5(0.3)	4.5(0.4)	0.681			
**Fasting Insulin** **(mU/ml; n = 18)**	11.5(9.0)	9.4(4.4)	8.5(4.0)	0.310			
**AUC glucose** **(mmol min/ml; n = 17)**	715.7(135.7)	765.8(115.9)	754.9(141.8)	0.171			
**AUC insulin** **(mU min/ml; n = 18)**	7752.0(3015.4)	8320.4(5383.7)	6727.3(4329.4)	0.005	0.841	0.010	0.002
**AUC C-peptide** **(nmol min/l; n = 18)**	217.4(76.6)	219.2(67.4)	193.0(63.7)	0.104			

Plasma lipids, glucose, insulin and C-peptide levels were assessed in fasting state. Second, third and fourth column contain average values and standard deviations for each of the regimes. The fifth column represents the level of significance for repeated measurements ANOVA. Column six to eight give the statistical significance for pairwise comparisons of the regimes (Least Significant Differences, p-values were not corrected for multiple testing). For pairwise comparing, p-values less than 0.017 were considered significant.

### Insulin Sensitivity

In six of 54 insulin and C-peptide curves and in 7 of 54 glucose curves one or two sample points were missing, these data were inputted using polynomial regression. In one glucose curve three sample points were missing, the remaining data were not used in the analyses.

**Figure 2:**
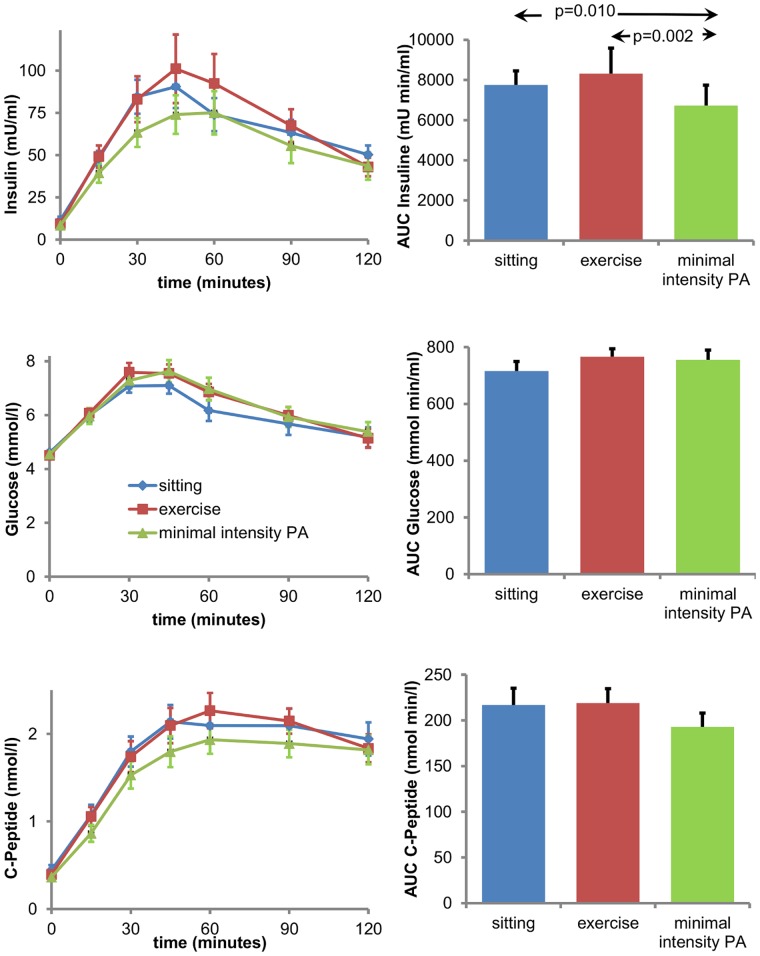
Patterns of insulin, glucose and C-peptide concentration during 2 h oral glucose tolerance test. 2a. Average insulin levels for each of the three regimes (blue: sitting, red: exercise, green: minimal intensity PA) during the oral glucose tolerance tests that were performed after each regime (left hand panel) and average area under the curve for each of the three regimes (right hand panel). Area under the curve was in the minimal intensity PA regime significantly smaller than in both other conditions. **2b.** Average glucose levels for each of the three regimes (blue: sitting, red: exercise, green: minimal intensity PA) during the oral glucose tolerance tests that were performed after each regime (left hand panel) and average area under the curve for each of the three regimes (right hand panel). **2c.** Average C-peptide levels for each of the three regimes (blue: sitting, red: exercise, green: minimal intensity PA) during the oral glucose tolerance tests that were performed after each regime (left hand panel) and average area under the curve for each of the three regimes (right hand panel). Abbreviations: PA, physical activity.

Insulin levels differed significantly between the regimes, insulin sensitivity index was nearly significant (p = 0.052). The ISI showed a trend for improvement after the minimal intensity PA regime. Pairwise comparison revealed that the AUC for insulin in the OGTT was significantly smaller after the minimal intensity PA regime than after the sitting (p = 0.010) and the exercise regime (p = 0.002; Table 2, Figure 2A). No major differences were observed in the glucose and C-peptide levels before and during the OGTT after each regime (Table 2, Figure 2B and 2C).

### Plasma Lipids

Triglycerides (p = 0.007), non-HDL cholesterol (p = 0.011) and apo B concentrations (p = 0.022) were significantly affected by the different regimes; pairwise comparison revealed that, in comparison to the sitting regime, these lipid measures were significantly reduced after the minimal intensity PA regime (with approximately 22%, 10% and 8%, respectively, Table 2). Triglycerides and non-HDL cholesterol showed a statistically, non-significant trend of improvement after the minimal intense PA condition compared to exercise. No effect of the exercise regime was observed compared to the sitting regime (Table 2). No major changes were observed in LDL-cholesterol, HDL-cholesterol and apo A–I.

Changes over conditions in triglycerides concentration and ISI did not correlate. Pearson’s correlation coefficient for changes in triglycerides and ISI between sitting and minimal intensity PA was −0.113 (p = 0.665); for the changes between sitting and exercise it was −0.388 (p = 0.112).

## Discussion

A sedentary lifestyle has become a major health threat in our affluent society [11]. Current guidelines on the prevention of cardiovascular disease promote at least ½ hr moderate to vigorous physical activity (MVPA) at least 5 days/week. They do not answer the question if, when DEE is held constant, such short bouts of exercise can compensate for the negative metabolic effects of inactivity. The present study, performed under free living conditions, suggests that 1 hour of daily physical exercise cannot compensate for the negative effects of inactivity on insulin sensitivity and plasma lipids if the rest of the day is spent sitting. Vice versa with nearly identical DEE reducing sitting time by walking/standing was more effective in improving insulin level and lipid parameters than 1 hour of moderate to vigorous bicycle exercise. This novel observation may have important health policy implications.

In the present study subjects were instructed during a run-in phase about the activity pattern and they received daily feedback. Subjects with a sedentary lifestyle were selected; both the ActivPAL data during the run-in phase and the questionnaires obtained at the end of the study suggested that the sitting regime reflected their daily activities. During the sitting regime they took approximately 4300 steps/day; less than 5000 steps/day is considered sedentary [18]. Participants followed the imposed regimes well, and as sleeping time was the same in the three regimes, estimated DEE was increased by 474 kcal during the exercise regime and slightly, but significantly, more with 553 kcal during the minimal intensity PA regime. As BMR was not measured, we cannot exclude that different activity regimes had different effects on BMR; this remains to be determined in future studies. Participants were instructed to consume the same caloric intake during the three regimes, and energy intake as well as meal composition were monitored with food diaries. Although such diaries are probably unreliable in absolute terms, participants probably did not alter their diet as no changes in energy intake and macronutrient composition were reported during the experimental conditions. Another limitation was that participants were not balanced over both sexes; the majority of subjects were females. Although, some studies have indeed shown sex differences in lipid metabolism, other authors did not show gender differences in insulin sensitivity or lipid metabolism in adaptation to physical activity. In previous bed rest studies inactivity led in both sexes to the development of insulin resistance [19] and resulted in similar effects on serum lipid and lipoprotein concentration for men and women [20]. In addition, Magkos et al. [21] showed that lipid metabolism was not influenced by menstrual cycle phase. Yeung et al. [22] reported change in insulin resistance over the menstrual cycle. In this study we did not control menstrual cycle. As the chance that menstrual phases match for all female participants similarly with the regime is small, it is unlikely that the menstrual cycle affected the results.

In line with earlier studies, we observed a positive, non-significant effect of physical exercise on triglycerides, non-HDL cholesterol and apo B as well as a (non-significant) 12% increase in insulin sensitivity. Physical exercise is currently seen as one of the cornerstones in the treatment of (sedentary) subjects with the metabolic syndrome and type 2 diabetes. However, MVPA seems to be a bridge too far for many of these subjects, due to lack of motivation, lack of time or physical impairments [12]. For instance, a 1 year walking programme had a drop-out rate of 65% in type 2 diabetic subjects [23]. The efficacy of such simple exercise programmes is hampered by the limitations caused by the underlying disease and by its complications. In a small population study in subjects with relatively well controlled type 2 diabetes we found that factors such as reduced muscle strength and diabetic neuropathy (present in 44% of the patients) were each associated with a reduction of the number of steps per day with approximately 30% [24]. Moreover, when healthy subjects (mean age of 59 years) performed daily exercises in line with current guidelines, total DEE remained unaltered as the increase in energy expenditure was compensated with longer periods of inactivity during the rest of the day [25]. These studies suggest that especially for groups at risk both low compliance and compensatory behaviour can compromise the effect of MVPA on health.

Several epidemiological studies suggest that too much inactivity is detrimental for health [5], [7], [26], [27], [28], [29], data from experimental, interventional inactivity studies are however scarce. Traditionally, bed rest studies have been performed as model of inactivity, and already 3 days of 24 hours bed rest can negatively affect insulin sensitivity [30], probably by disrupting muscle insulin signaling, and can result in a rise of fasting triglycerides [31]. However, it is questionable whether strict bed rest is a valid model for a sedentary lifestyle. An increase in sitting time for 2 weeks resulted in impaired peripheral insulin sensitivity in healthy volunteers [8]. However, it was not clear to which extent a positive energy balance contributed to this effect. This was addressed in a recent study, in which physical activity was reduced during 1 day in healthy volunteers, resulting in a decrease in DEE of approximately 800 kcal, with and without balancing energy intake. Although the largest effect was seen after a positive energy balance, insulin action was also impaired after inactivity when energy expenditure and intake were in balance [9]. However, this latter study did not address the question on how inactivity should be reduced, by a relative short bout of physical exercise or by substituting sitting with light intensity activities such as standing and walking at a leisurely pace. Recently, Dunstan et al. [32] showed that interrupting sitting time independent of the intensity of walking performed during the breaks had positive acute, effects on postprandial glucose and insulin levels, but in this study energy expenditure was not controlled. It cannot be concluded whether this positive effect of breaking sitting time is caused by the reduction of the sitting time or by the increased energy expenditure that concurred.

To our knowledge this is the first study that separately manipulated sitting time, physical exercise and DEE in healthy, but sedentary subjects and the novel finding was that a 1 hour bout of physical exercise cannot completely compensate for the negative effects of inactivity on insulin, triglycerides, apo B and non-HDL cholesterol levels. From a traditional exercise physiological point of view, the results of this study might appear surprising; walking at a leisurely pace and standing were more effective than a high intensity physical exercise alternative. As argued by Hamilton et al. [33], we seem to have forgotten to ask ‘what does *in*activity do?’. The human body not only adapts to exercise initiated stresses, but as our results underline, it also reacts to inactivity, that is increasingly becoming the dominant lifestyle in westernized societies. In addition, this study underlines the importance of using strict definitions of terms like ‘sedentary’, ’inactivity’, ‘active’, ‘sports’ and ‘exercise’ [34], [35]. With respect to insulin and plasma lipid levels ‘not participating in exercise or sports’ does not necessarily have an identical effect as ‘being sedentary’, and oppositely ‘to exercise daily’ does not exclude a ‘sedentary lifestyle’ with negative metabolic effects, as shown in this study. Given the results of the present study it is questionable whether replacing sitting by a daily bout of exercise would be desirable in sedentary subjects with the metabolic syndrome or with type 2 diabetes. This remains to be determined in further studies. The 22000 steps/day that concurred with the minimal intensity PA regime seem to be quite a challenge, if it comes to implementation in daily living. Future studies need to explore the dose-response relation of minimal intensity PA.

Reducing sitting time with approximately 6 hours resulted in this study in a marked 15% reduction in insulin levels and a non-significant 11% reduction in C-peptide levels. The lack of statistically significant differences in C-peptide levels was probably caused by a lack of statistical power due to the variability of the responses to the OGTT, as discussed above. Moreover, in comparison to the sitting regime, triglycerides, non-HDL-cholesterol and apo B levels were 22%, 10% and 8% lower during minimal intensity PA. How, i.e. by which mechanism, inactivity and minimal intensity PA affect insulin sensitivity and plasma lipids remains to be determined. Given the short duration of each (in)activity regime in our study, changes in microvascular perfusion or mitochondrial function seem less likely. The reduction in triglycerides is compatible with a beneficial effect of minimal intensity PA on free fatty acids (FFA) clearance and/or lipid oxidation and impaired lipid oxidation is thought to be one of the fundamental steps in inactivity induced insulin-resistance [31]. Adenosine monophosphate-activate protein kinase (AMPK) plays an important role in both insulin signaling and FFA oxidation, it is stimulated by muscle contractions and loss of AMPK activity might therefore be one of the detrimental consequences of inactivity [10]. Another possible mechanism underlying the changes in triglycerides might be a change in lipoprotein lipase (LPL) activity. As reviewed elsewhere, inactivity induces a decrease in LPL levels which can result in a blunted plasma triglyceride uptake; minimal intensity PA instead increases LPL activity and hereby increases triglyceride cellular uptake [33]. Indeed in our participants the greatest differences were found in triglyceride plasma levels and a decrease in LPL activity due to a prolonged sitting time may thus be –at least partially- responsible for the higher triglyceride levels in both the sitting and the exercise regimes.

In previous exercise studies, the activities during the rest of the day were often not controlled, in the present study we strictly controlled (in)activity behaviour 24 hr/day. However, the duration of the interventions in the present study was relatively short (4 days) and in future studies the effects of the duration of inactivity need to be addressed, preferably also over longer periods. Moreover, more detailed assessment of insulin sensitivity, such as hyperinsulinemic euglycemic clamp techniques, should be used to unravel the underlying mechanisms.

### Conclusions

One hour of daily physical exercise cannot compensate for the negative effects of inactivity on insulin sensitivity and plasma lipids if the rest of the day is spent sitting. Reducing inactivity by low intensity activities such as walking at a leisurely pace and standing is more effective than physical exercise in improving these parameters in sedentary subjects. Our study suggests that in addition to health interventions that stress the importance of spending enough energy to maintain a neutral energy balance, a minimal daily amount of non-sitting time should also be promoted.
