# Correction: Minimal Intensity Physical Activity (Standing and Walking) of Longer Duration Improves Insulin Action and Plasma Lipids More than Shorter Periods of Moderate to Vigorous Exercise (Cycling) in Sedentary Subjects When Energy Expenditure Is Comparable

**DOI:** 10.1371/journal.pone.0105135

**Published:** 2014-08-05

**Authors:** The PLOS ONE Staff

There is an error in the third sentence of the Methodology/Principle Findings section of the Abstract. The correct sentence is: The sitting and exercise regime had comparable numbers of sitting hours; compared to the exercise regime, the minimal intensity PA regime had a higher estimated daily energy expenditure (238kcal/day).

There is an error in the sixth sentence of the Methodology/Principle Findings section of the Abstract. The correct sentence is: In the sitting regime, daily energy expenditure was about 300 and 500 kcal lower than in the exercise and minimal intensity PA regimes respectively.

There is an error in the second sentence of the Physical activity, postural allocation and energy intake and expenditure segment of the Results section. The correct sentence is: During the exercise regime all participants had a daily, 1 hour bicycle exercise with a mean increase of heart rate of 52 ± 3 beats/min, resulting in an estimated energy expenditure of 367 ± 5 kcal.

There is an error in the penultimate sentence of the Physical activity, postural allocation and energy intake and expenditure segment of the Results section. The correct sentence is: Compared to the sitting regime, estimated DEE was about 300 and 500 kcal higher during the exercise and minimal intensity PA regimes respectively; estimated DEE was 238 kcal/day higher during the minimal intensity PA in comparison to the exercise regime: 2248 vs 2486 kcal/day (p<0.001).

There is an error in the fourth sentence of the second paragraph in the Discussion section. The correct sentence is: Participants followed the imposed regimes well, and as sleeping time was the same in the three regimes, estimated DEE was increased by 314 kcal during the exercise regime and with 553 kcal during the minimal intensity PA regime.

There is an error in Table 2. In the Daily Energy Expenditure row, the data is incorrect in the Exercise regime column and the p exerc vs MIPA column. Please see the corrected table below.

**Table 2:** Daily energy intake and expenditure, time spent in activity categories and glucose metabolism and plasma lipids.

	Sitting regime	Exercise regime	Minimal intensity PA regime	p-value	p sit vs exerc.	p sit vs MIPA	p exerc vs MIPA
**Daily Energy intake (kcal, n = 18)**	1539(427)	1477(352)	1394(292)	0.136			
**Proteins (g, n = 18)**	61.1(14.8)	59.7(13.5)	55.6(13.4)	0.165			
**Fat (g, n = 18)**	54.5(14.7)	50.2(19.6)	50.1(12.2)	0.248			
**Carbohydrates (g, n = 18)**	199.0(68.9)	196.7(48.9)	180.0(51.2)	0.227			
**Daily Energy Expenditure (kcal, n = 16)**	1934(88)	2248(93)	2486(121)	<0.001	<0.001	<0.001	<0.001
**Sitting time (hr, n = 17)**	13.6(1.2)	12.7(1.7)	7.4(1.3)	<0.001	0.002	<0.001	<0.001
**Standing time (hr, n = 17)**	0.99(0.50)	1.08(0.48)	3.08(0.88)	<0.001	0.166	<0.001	<0.001
**Active-not exercise time (hr, n = 17)**	0.81(0.29)	1.01(0.26)	4.85(0.63)	<0.001	0.001	<0.001	<0.001
**Sleeping time (hr, n = 17)**	8.58(0.74)	8.17(1.37)	8.65(0.93)	0.200			
**Steps/day (n = 16)**	4324(1485)	6049(1402)	27590(3724)	<0.001	<0.001	<0.001	<0.001
**Triglycerides (mmol/l; n = 18)**	0.90(0.26)	0.85(0.35)	0.70(0.23)	0.007	0.326	0.002	0.029
**Total Cholesterol (mmol/l; n = 18)**	4.20(0.67)	4.11(0.60)	3.96(0.50)	0.171			
**HDL-Cholesterol (mmol/l; n = 18)**	1.26(0.34)	1.27(0.28)	1.30(0.30)	0.686			
**Non-HDL-Cholesterol (mmol/l; n = 18)**	2.94(0.47)	2.84(0.57)	2.65(0.48)	0.011	0.275	0.007	0.048
**LDL-Cholesterol (mmol/l; n = 18)**	2.53(0.51)	2.45(0.57)	2.34(0.49)	0.094			
**Apo A-I (g/l; n = 18)**	1.57(0.24)	1.57(0.21)	1.55(0.21)	0.905			
**Apo B (g/l; n = 18)**	0.75(0.12)	0.70(0.16)	0.69(0.14)	0.022	0.052	0.005	0.627
**Insulin Sensitivity Index (n = 17)**	20.4(8.2)	22.8(9.9)	26.3(11.7)	0.052	0.246	0.051	0.036
**Fasting Glucose (mmol/ml; n = 17)**	4.6(0.4)	4.5(0.3)	4.5(0.4)	0.681			
**Fasting Insulin (mU/ml; n = 18)**	11.5(9.0)	9.4(4.4)	8.5(4.0)	0.310			
**AUC glucose (mmol min/ml; n = 17)**	715.7(135.7)	765.8(115.9)	754.9(141.8)	0.171			
**AUC insulin (mU min/ml; n = 18)**	7752.0(3015.4)	8320.4(5383.7)	6727.3(4329.4)	0.005	0.841	0.010	0.002
**AUC C-peptide (nmol min/l; n = 18)**	217.4(76.6)	219.2(67.4)	193.0(63.7)	0.104			

Plasma lipids, glucose, insulin and C-peptide levels were assessed in fasting state. Second, third and fourth column contain average values and standard deviations for each of the regimes. The fifth column represents the level of significance for repeated measurements ANOVA. Column six to eight give the statistical significance for pairwise comparisons of the regimes (Least Significant Differences, p-values were not corrected for multiple testing). For pairwise comparing, p-values less than 0.017 were considered significant.
